# 

**Published:** 1938

**Authors:** 


### CONTENTS.

| | Page |
|---|---|
| **Medical Defence.** By Clifford A. Moore, M.S., Ch.M., F.R.C.S. | 1 |
| **Some Clinical Applications of the Male and Female Sex Hormones.** By G. L. Foss, M.B., B.Ch. .. .. .. | 23 |
| **Thoracic Surgery.** By A. L. d'Abreu, Ch.M., F.R.C.S. .. .. | 43 |
| **A Case of Lobectomy for Bronchiectasis.** By A. Wilfrid Adams, M.S., F.R.C.S. .. .. .. .. .. .. | 63 |
| **Reviews of Books** .. .. .. .. .. .. .. | 67 |
| **Editorial Notes** .. .. .. .. .. .. .. .. | 78 |
| **Obituary:** John Courtenay MacWatters, M.B.E., M.R.C.S., L.R.C.P. .. .. .. .. .. .. .. .. .. | 83 |
| **Meetings of Societies** .. .. .. .. .. .. .. | 85 |
| **The Medical Library of the University of Bristol** .. .. | 86 |
| **Publications Received** .. .. .. .. .. .. .. | 90 |
| **Local Medical Notes** .. .. .. .. .. .. .. | 91 |

